# Ankle joint pressure change in varus malalignment of the tibia

**DOI:** 10.1186/s12891-020-3163-2

**Published:** 2020-03-04

**Authors:** Yuan Zhu, Xingchen Li, Xiangyang Xu

**Affiliations:** 1 Department of Orthopaedics, Ruijin Hospital, Shanghai Jiaotong, University School of Medicine, Shanghai, China; 2 Department of Orthopaedics, Ruijin Hospital North, Shanghai Jiaotong, University School of Medicine, Shanghai, China

**Keywords:** Ankle biomechanics, Tibial varus, Joint pressure measurement

## Abstract

**Background:**

Varus malalignment of the tibia could alter ankle biomechanics, and might lead to degenerative changes of the ankle joint. However, previous studies failed to report the detailed changes of ankle biomechanics in varus malalignment of the tibia. The aim of this biomechanical study was to evaluate how the ankle joint pressure would change in response to the incremental increases in varus malalignment of the tibia.

**Methods:**

Eight fresh-frozen human cadaver legs were tested in this study. Varus malalignment of the tibia and a total of 600 N compressive force was simulated using a custom made fixture. Intra-articular sensors (TeckScan) were inserted in the ankle joint to collect the ankle joint pressure data. The testing sequence was 0°, 2°,4°,6°,8°,10°,12°,14°,16°,18°,20° of tibial varus.

**Results:**

As the tibial varus progressed, the center of force (COF) shifted laterally both for the medial and lateral aspect of the ankle joint. For the medial aspect of the ankle joint, the lateral shift reached its maximum at 8° [2.76 (1.62) mm, *p* = 0.002] of tibial varus, while for the lateral aspect of the ankle joint, the lateral shift reached its maximum at 12° [2.11 (1.19) mm, *p* = 0.002] of tibial varus. Thereafter, the COF shifted medially as the tibial varus progressed. For the lateral aspect of the ankle joint, The P_mean_ increased from 2103.8 (625.1) kPa at 0° to 2295.3 (589.7) kPa at 8° of tibial varus (*p* = 0.047), significant difference was found between the P_mean_ at 0° and 8° (*p* = 0.047) of tibial varus. Then as the tibial varus progressed, the P_mean_ decreased to 1748.9 (467.2) kPa at 20° of tibial varus (*p* = 0.002). The lateral joint pressure ratio also increased from 0.481 (0.125) at 0° to 0.548 (0.108) at 10° of tibial varus (*p* = 0.002), then decreased to 0.517 (0.101) at 20° of tibial varus (*p* = 0.002) .

**Conclusions:**

For mild tibial varus deformities, there was a lateral shift of COF and lateral stress concentration within the ankle joint. However, as the tibial varus progressed, the COF shifted medially and the lateral stress concentration decreased.

## Background

The alignment of the lower extremity is critical for orthopaedic surgeons, both for preoperative and postoperative evaluation. It is believed that the varus or valgus deformity of the lower extremity is highly associated with osteoarthritis, especially the knee joint [1–4]. Previous study demonstrated that the knee joint alignment and kinematics was altered in patients with early knee ostoearthritis [5]. Patient with varus knee often had a varus inclined tibia, which could subsequently alter normal biomechanics of the ankle joint. It was thought that varus tibia can lead to stress concentration on the medial aspect of the ankle joint, prolonged stress will result in degenerative changes of the cartilage within the ankle joint [6].

Norton and colleagues [7] studied the compensatory mechanism of hindfoot for advanced knee osteoarthritis, they found that as the mechanical axis angle became either more varus or valgus, the hindfoot would subsequently orient in more valgus or varus position in order to maintain a normal mechanical alignment of the lower extremity. Therefore, for patients with varus malalignment of the tibia, would the compensatory mechanism further affect the ankle joint biomechanics? Ting and colleagues [8] found that anterior and posterior bow deformities produced a greater change in contact area of the tibiotalar joint than with valgus or varus deformities. The subtalar joint might have compensated for varua and valgus deformities. The PreScale TM pressure-sensitive film was utilized for the measurement of contact area and pressure, and 0°, 5°, 10°, 15° of deformities was simulated. They failed to report the detailed ankle joint pressure change in response to the incremental increases in varus malalignment of the tibia.

The aim of this biomechanical study was to investigate the detailed ankle joint pressure change as the tibial varus progressed. We hypothesized that the pressure on the lateral aspect of the ankle joint will increase as the result of valgus inclination of the subtalar joint in response to the varus tibia malalignment. As the varus deformity progressed, the valgus inclination of the subtalar joint would reach its’ maximum and then fail, thereafter, the subtalar joint would convert into a varus inclination resulting a sudden medial stress concentration.

## Methods

A total of 8 fresh-frozen human cadaver legs were utilized for the biomechanical testing, the specimens were thawed to room temperature (24 °C). The mean age of the cadavers was 71.4 (7.4) years old, 4 of the 8 were men and 4 of the 8 were right foot. X-rays were obtained for each specimen and none of them had malalignment of the tibia, hindfoot, nor preexisting subtalar joint osteoarthritis. All specimens had normal range of motion of both ankle and subtalar joint. The anterior soft tissue (including skin, subcutaneous tissue, anterior joint capsule, tendons and neurovascular bundles) of the ankle joint were dissected for the access to the ankle joint. Both the medial and lateral ankle ligaments were well preserved.

The tibia and fibula was cut at 20 cm above the ankle joint. For each specimen, the proximal tibia and fibula was potted securely into a custom made shell, then mounted on a custom made fixture. The tibia and fibula was embedded and securely fixed into the shell using dental gypsum. Load was applied to the tibia and fibula via the custom made shell. Each specimen must be potted in neutral position, no plantarflexion or dorsiflexion of the ankle joint in sagittal plane, no varus or valgus malalignment of the hindfoot in coronal plane, no internal or external rotation of the foot in horizontal plane.

**Fig. 1:**
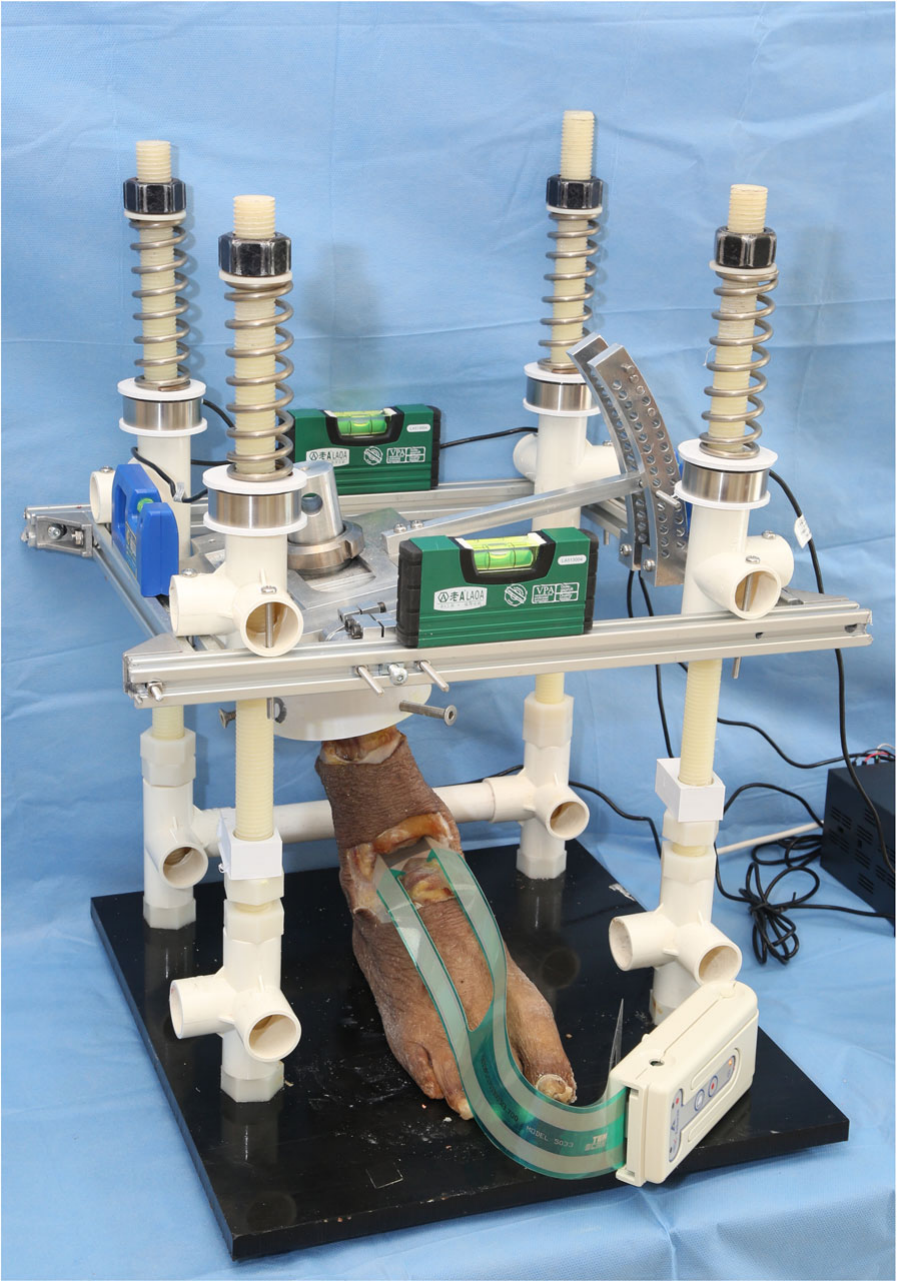
The custom made fixture, the varus malalignment of the tibia was simulated by inserting the bolt into specific holes

The custom designed fixture was utilized for the testing. Spirit levels were utilized to make sure both the working table and the top plate was horizontal throughout the entire testing process. The varus malalignment of tibia (0°,2°,4°,6°,8°,10°,12°,14°,16°,18°,20° of tibial varus) was simulated by a custom made apparatus. Each hole on the apparatus represented a specific varus angle. A bolt was used to fix the specimen at a desired varus angle (Fig. 1).

The four threaded polyethylene pillars were utilized to connect the top plate, the compressive forces were exerted via the four pillars. Sensor cells were placed in each pillar, a monitor was connected to each sensor cell, displaying the real-time force. Springs were placed right above each force sensor, then followed by nuts, compressive forces could be generated by twisting the nut on the spring.

**Fig. 2:**
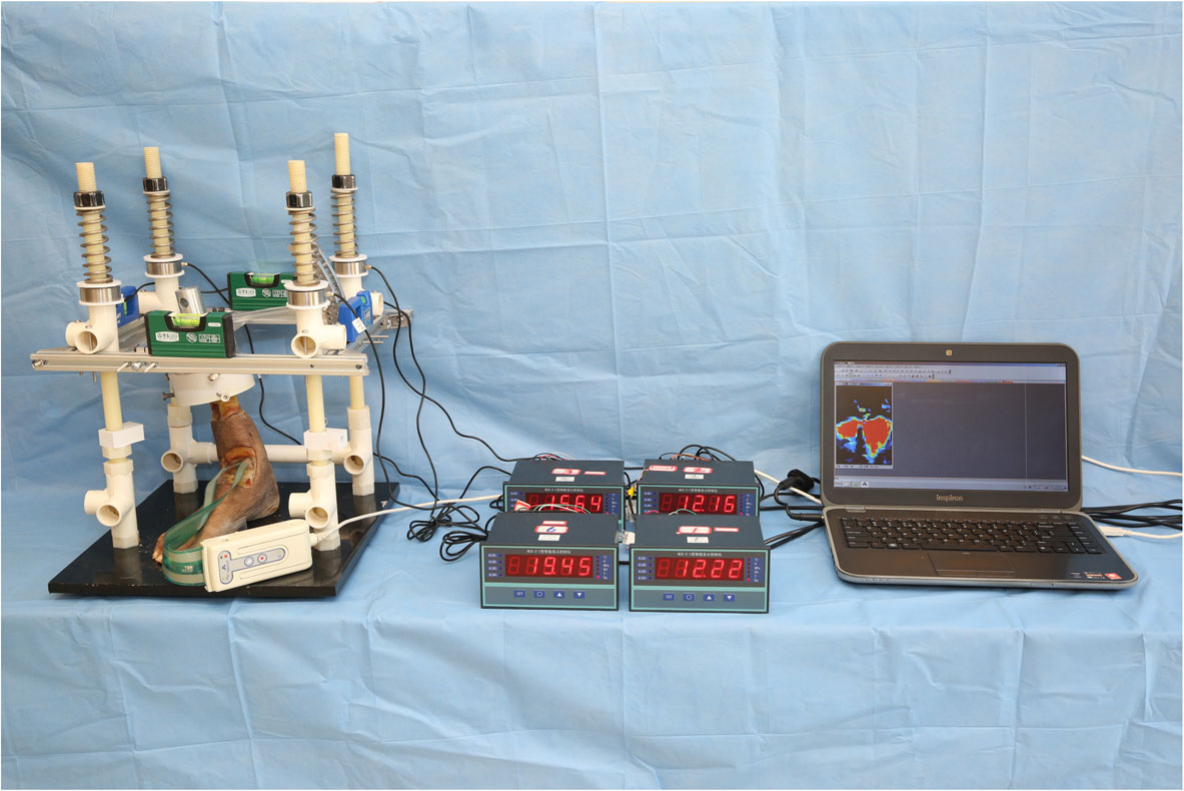
The specimen was mounted in the custom made fixture, the force applied on each pillar could be read on the screen and the real-time joint pressures were collected

The sensor pads (Model 6900, TekScan, Inc., South Boston, MA), with each pad measuring 14*14 mm, each pad had 121 senels (11*11 sensels), the column and row spacing was 1.3 mm, resulting in a spatial resolution of 0.62 mm^2^ per sensel. Two pads were put side by side within the ankle joint for the measurement of the ankle joint pressure. The sensor pads were inserted into the ankle joint from anterior and secured by thumbtacks to the distal tibial metaphysis and the foot in order to avoid sensor motion during testing [9, 10]. The sensor pads were connected to the handle which could be further connected to a personal computer, data including pressure, force was collected using I-Scan software (Fig. 2).

The baseline ankle joint pressure distribution was initially collected for each specimen. The specimen was fixed at 0° of tibial varus by inserting a bolt, the foot was then placed onto the floor freely. A compressive force was generated through the 4 pillars by twisting the nuts. Making sure the top plate was maintained horizontally throughout the testing. A 600 N compressive force was applied to simulate the normal load within the ankle joint during ambulation. Both the medial and lateral ankle joint pressure data was collected. Then free the specimen by removing the bolt and then the top plate of the fixture was lifted upward, followed by 2° of tibial varus, then 4°,6°,8°,10°,12°,14°,16°,18°,20°. Both the medial and lateral aspect of the ankle joint pressure data were collected for each alignment.

### Statistical analysis

SPSS V.23 software (IBM Inc., New York) was used for the data analysis. Matlab was used for the calculation of the center of force (COF) shift, the peak pressure (P_max_) and the mean pressure (P_mean_). The lateral shift of COF relative to the ankle joint was defined as positive, the medial shift was defined as negative. S-W test was used for the test of normality. Paired student t test was utilized to determine the significant differences for the COF shift, P_max_ and P_mean_ at different working conditions. The level of significance was set to a *p* value < 0.05.

## Results

### The COF shift

**Fig. 3:**
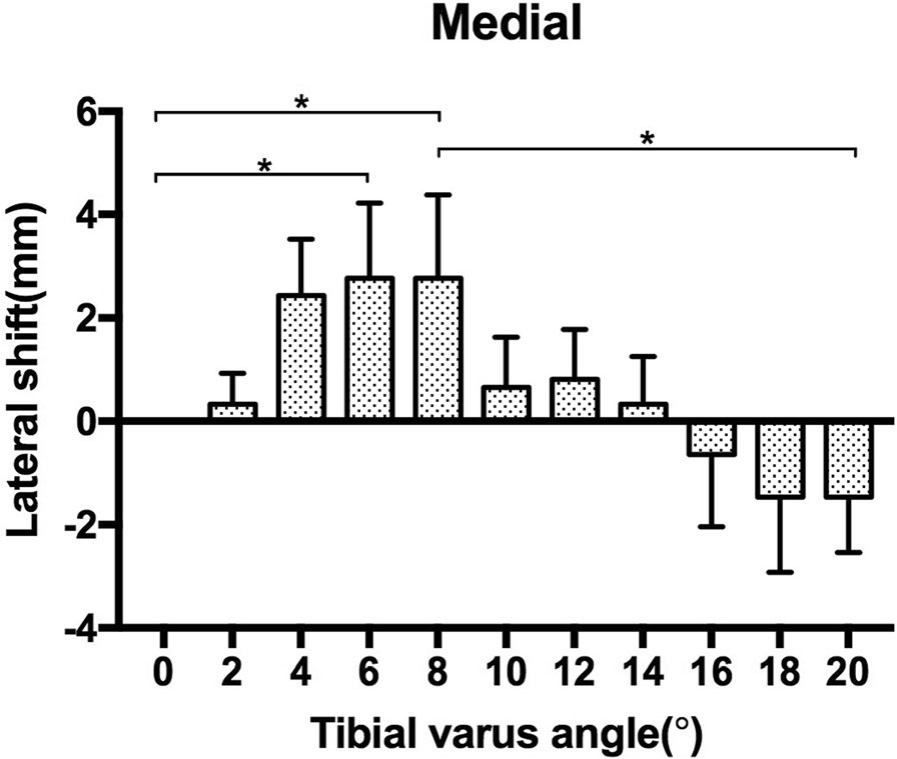
The lateral shift of medial COF as the tibial varus deformity progressed. The asterisk(*) denotes significant difference

**Fig. 4:**
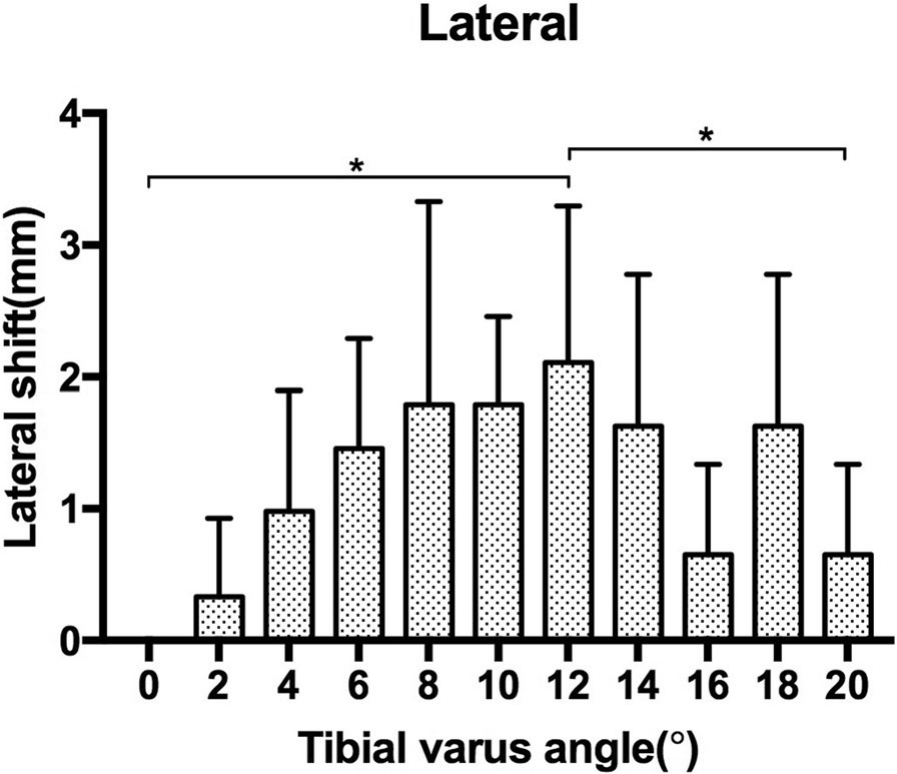
The lateral shift of lateral COF as the tibial varus deformity progressed

As the tibial varus progressed from 0° to 20° of tibial varus, the COF shifted laterally both for the medial and lateral aspect of the ankle joint initially, then the COF converted to shift medially as the tibial varus progressed to 20°. The maximum lateral shift of the medial COF was noticed at 8° [2.76 (1.62) mm, *p* = 0.002] of tibial varus. Then the COF shifted medially as the tibial varus progressed, the lateral shift of the medial aspect COF was − 1.46 (1.08) mm at 20° of tibial varus, significant difference was found between 8° and 20° of tibial varus, *p* = 0.001 (Fig. 3). While the maximum lateral shift of the lateral COF was noticed at 12° [2.11 (1.19) mm, *p* = 0.002] of tibial varus, then it converted to shift medially as the tibial varus progressed from 12° to 20°. The lateral shift for the lateral aspect of COF was 0.65 (0.69) mm at 20° of tibial varus, significant difference was found between 12° and 20° of tibial varus, *p* = 0.026 (Fig. 4) (Table 1).

**Table 1:** The COF shift, P_max_, P_mean_ and the lateral joint pressure ratio change in response to the series of tibial varus

		0° varus	2° varus	4° varus	6° varus	8° varus	10° varus	12° varus	14° varus	16° varus	18° varus	20° varus
The COF shift (Medial)	Mean(SD)(mm)	0(0)	0.33(0.60)	2.44(1.08)	2.76(1.46)	2.76(1.62)	0.65(0.98)	0.81(0.97)	0.33(0.92)	−0.65(1.39)	− 1.46(1.46)	−1.46(1.08)
	0° varus	p value	0.17	0	0.001	0.002	–	–	–	–	–	–
	8° varus	p value	–	–	–	–	0.048	0.02	0.002	0.003	0	0.001
The COF shift (Lateral)	Mean(SD)(mm)	0(0)	0.33(0.60)	0.98(0.92)	1.46(0.83)	1.79(1.54)	1.79(0.67)	2.11(1.19)	1.63(1.15)	0.65(0.69)	1.63(1.15)	0.65(0.69)
	0° varus	p value	0.17	0.02	0.002	0.014	0	0.002	–	–	–	–
	12° varus	p value	–	–	–	–	–	–	0.285	0.026	0.197	0.026
The Pmax (Medial)	Mean(SD)(kPa)	5303.4(1105.1)	4973.9(986.0)	4883.5(841.7)	4939.4(959.7)	4751.8(741.5)	4708.8(898.2)	4710.9(959.7)	4615.8(791.3)	4464.1(884.4)	4268.9(752.2)	4432.1(948.6)
	0° varus	p value	0.032	0.219	0.179	0.148	0.148	0.209	0.102	0.081	0.039	0.045
The Pmax (Lateral)	Mean(SD)(kPa)	5135.5(1095.3)	4754.7(1205.3)	4727.8(694.9)	4940.1(804.1)	5000.8(761.1)	4844.5(756.7)	4412.6(581.9)	4478.0(238.0)	4525.5(525.6)	4324.1(866.8)	4279.4(666.9)
	0° varus	p value	0.022	0.087	0.362	0.691	0.439	0.085	0.096	0.18	0.13	0.116
The Pmean (Medial)	Mean(SD)(kPa)	2266.4(672.7)	2107.6(654.9)	2064.8(632.7)	2064.4(624.5)	2027.6(632.9)	1891.2(563.9)	1895.4(499.5)	1802.2(481.1)	1742.5(314.6)	1587.1(453.6)	1618.7(421.5)
	0° varus	p value	0.021	0.072	0.056	0.089	0.007	0.018	0.004	0.10	0.007	0.006
The Pmean (Lateral)	Mean(SD)(kPa)	2103.8(625.1)	2059.7(465.6)	2109.3(510.8)	2183.7(581.3)	2295.3(589.7)	2280.7(519.3)	2043.6(456.3)	2053.0(416.6)	1972.1(481.0)	1864.5(449.2)	1748.9(467.2)
	0° varus	p value	0.555	0.952	0.499	0.047	–	–	–	–	–	–
	8° varus	p value	–	–	–	–	0.776	0.014	0.015	0.012	0.01	0.002
The lateral joint pressure ratio	Mean(SD)	0.481(0.12)	0.499(0.11)	0.508(0.11)	0.515(0.11)	0.531(0.11)	0.548(0.11)	0.519(0.10)	0.534(0.10)	0.527(0.09)	0.540(0.10)	0.517(0.10)
	0° varus	p value	0.11	0.122	0.088	0.011	0.002	–	–	–	–	–
	10° varus	p value	–	–	–	–	–	0.014	0.191	0.096	0.405	0.002

### The P_max_

As the tibial varus progressed from 0° to 20° of tibial varus, it seemed that the P_max_ for both the medial and lateral aspect of the ankle joint decreased gradually. It was 5303.4 (1105.1) kPa at 0° and 4432.1 (948.6) kPa at 20° of tibial varus for the medial aspect of the ankle joint, significant difference was found for the medial P_max_ at 0° and 2° (*p* = 0.032), 0° and 18° (*p* = 0.039), 0° and 20° (*p* = 0.045) of tibial varus. However, it was 5135.5 (1095.3) kPa at 0° and 4279.4 (666.9) kPa at 20° of tibial varus for the lateral aspect of the ankle joint, significant difference was only found for the lateral P_max_ at 0° and 2° of tibial varus, *p* = 0.022. (Table 1).

### The P_mean_

**Fig. 5:**
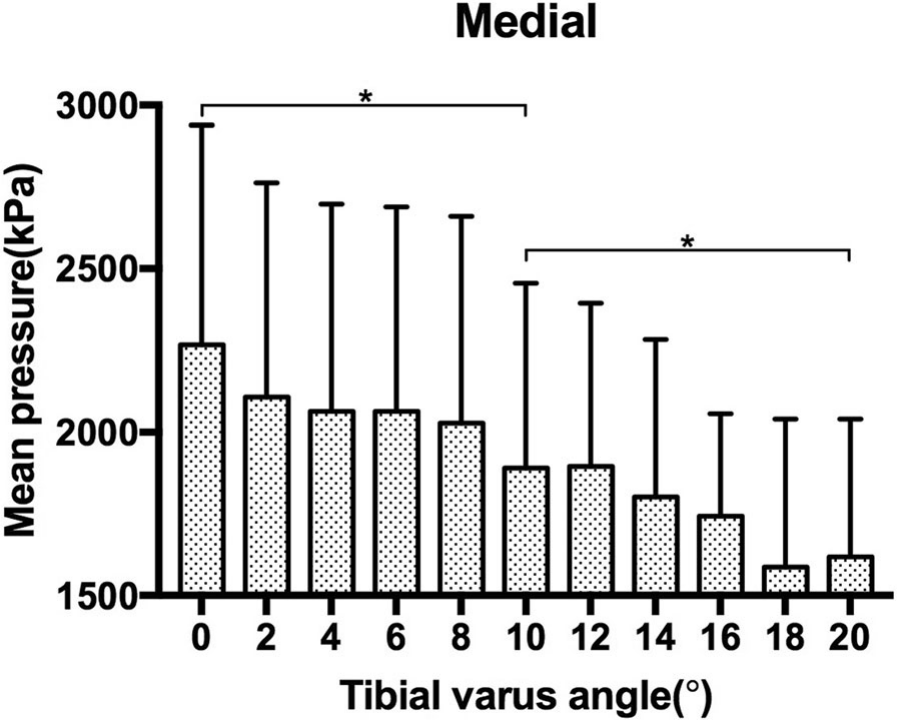
The mean pressure of the medial aspect of the ankle joint as tibial varus progressed

**Fig. 6:**
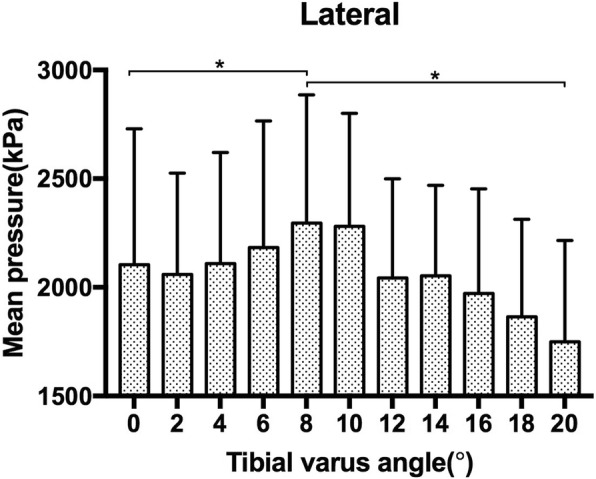
The mean pressure of the lateral aspect of the ankle joint as tibial varus progressed

The P_mean_ for the medial aspect of the ankle joint was 2266.4 (672.7) kPa at 0°, 1891.2 (563.9) kPa at 10° and 1618.7 (421.5) kPa at 20° of tibial varus. When compared with the baseline mean pressure at 0°, significant differences were found for both 10° (*p* = 0.007) and 20° (0.006) of tibial varus. The P_mean_ for the medial aspect of the ankle joint decreased as the tibial varus progressed from 0° to 20° of tibial varus (Fig. 5). However, for the lateral aspect of the ankle joint, the P_mean_ increased gradually from 0° to 8° of tibial varus, it was 2103.8 (625.1) kPa at 0° and 2295.3 (589.7) kPa at 8° of tibial varus, significant difference was found between the P_mean_ at 0° and 8° (*p* = 0.047) of tibial varus. Then as the tibial varus progressed from 8° to 20°, it converted to decrease and plummeted to 1748.9 (467.2) kPa at 20° of tibial varus, significant difference was found between the P_mean_ at 8° and 20° (*p* = 0.002) of tibial varus (Fig. 6) (Table 1).

### The lateral joint pressure ratio

**Fig. 7:**
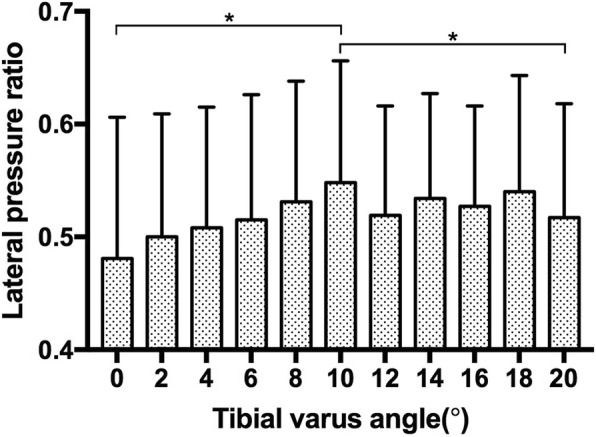
The lateral joint pressure ratio as the tibial varus progressed

In order to investigate the joint pressure change on the medial and lateral aspect of the ankle joint, we introduced the lateral joint pressure ratio, it was a ratio of the lateral mean pressure divided by the whole joint mean pressure. It seemed that the lateral joint pressure ratio increased from 0° to 10° of tibial varus, it was 0.481 (0.125) at 0° and 0.548 (0.108) at 10° of tibial varus, significant difference was found between these two conditions (*p* = 0.002). As the tibial varus continued to progress from 10° to 20°, it seemed that the lateral joint pressure ratio decreased. It was 0.517 (0.101) at 20° of tibial varus, significant difference was found between these two conditions (*p* = 0.002) (Fig. 7) (Table 1).

## Discussion

It had been widely recognized that varus ankle deformity was associated with varus type ankle osteoarthritis [11–13]. We assumed that the prolonged exposure to eccentric loading on the medial aspect of the ankle joint will result in stress concentration and the continuous eccentric loading may develop degenerative changes of the cartilage and lead to osteoarthritis eventually.

However, according to our biomechanical study, we failed to find any evidence for medial stress concentration as the tibial varus progressed to 20°. The P_max_ on both the medial and lateral aspect of the ankle joint decreased as the tibial varus progressed from 0° to 20°. And so did the P_mean_ for the medial aspect of the ankle joint, from 2266.4 (672.7) kPa at 0° to 1618.7 (421.5) kPa at 20° of tibial varus. Oneda and colleagues [14] reported a similar result, they performed a biomechanical investigation using a rigid-body spring model and concluded that varus deformity of the distal joint surface of tibia by itself does not result in medial stress concentration in the ankle. Why the opposite was observed?

Hayashi and colleagues [15] retrospectively reviewed 133 ankles in 80 patients (OA group) and 62 ankles in 50 subjects (control group). The weightbearing X-rays were investigated, and they concluded that the valgus inclination of the subtalar joint progressed until the intermediate stage and converted to varus position at the later stage. Wang and colleagues [16] retrospectively investigated the X-rays of 233 ankles in 226 patients and 60 ankles from 60 subjects. They concluded that the subtalar joint often compensated for the malaligned ankle in static weightbearing, however, the compensatory mechanism often relied on the health condition of the subtalar joint, the healthier the subtalar joint, the more it could compensate for the malaligned ankle joint. Krahenbuhl and colleague [17] retrospectively investigated subtalar joint alignment of 88 patients and 27 healthy volunteers in different stages of ankle OA using weightbearing CT scans. A more valgus subtalar joint alignment was found in patients with varus ankle OA, compensation did not correlate with the stage of ankle OA.

These studies also demonstrated that the subtalar joint does compensate for the varus ankle deformity. However, they failed to explain how this compensatory mechanism happened and whether this compensation would alter the joint pressure distribution within the ankle joint. In this biomechanical test, we found a lateral shift of COF and lateral stress concentration for mild varus deformities. As the tibial varus progressed from 0° to 20°, the COF for both medial and lateral aspect of the ankle joint shifted laterally till around 10° of tibial varus, and then shifted medially as the tibial varus progressed. The mean pressure of the lateral aspect of the ankle joint increased till about 10° of tibial varus, and then converted to decrease as the tibial varus continued to progress. This phenomenon could be explained by the compensation of the subtalar joint. The subtalar joint played a significant role in maintaining the talus in its normal relationship to the tibia, it acted as a torque transmitter and compensates for tibial varus deformities [7, 8, 18]. As calcaneus was in contact with the ground, the subtalar joint would compensate for the varus tilted ankle joint in order to maintain a normal hindfoot alignment. This valgus inclination of calcaneus might result in stress concentration on the lateral aspect of ankle joint and lateral shift of the COF as well. In addition, subtalar joint restriction could result in a decrease in lateral tibiotalar contact caused by the inhibition of calcaneal eversion [8]. So our biomechanical study favored these studies, the subtalar joint might have compensated for deformity above the ankle joint. According to Hayashi and colleagues [15], they found that the valgus inclination of subtalar joint will convert into varus inclination as the varus ankle deformity increase, because of the failure of subtalar joint compensation. However, in this study, we failed to find a sudden medial stress concentration even with a 20° of tibial varus. Further studies should be needed to explore whether the medial stress concentration would occur as the deformity reached greater than 20° of tibial varus.

There are some limitations for this study. First of all, this is a preliminary study which only included the joint pressure data, the compensatory mechanism of the subtalar joint should be supported by adding the radiological data of both the ankle and subtalar joint (including CT data and weightbearing X-rays). Further studies are needed to fully understand the compensatory mechanism of the subtalar joint. Second limitation is that this is a static biomechanical study, the ankle and subtalar joint biomechanics might act differently in vivo, we also neglected the tendon forces around the ankle joint in this study.

## Conclusions

We found a lateral shift of the COF and lateral stress concentration for mild tibial varus. However, as the tibial varus continued to progress, the COF shifted medially and the lateral stress concentration decreased. This phenomenon might be explained by the valgus inclination of subtalar joint in compensation for the varus tibial malalignment. However, further biomechanical studies are needed to fully understand the compensatory mechanism of the subtalar joint.

## Data Availability

All the data supporting our findings was contained within the manuscript. And all data in this study was freely available to any researcher for noncommercial purposes.

## Abbreviations

COF: Center of force
OA: Osteoarthritis
